# Centering Birthing Experiences of Women of Color: Protocol for a Qualitative Maternal Near Miss Study

**DOI:** 10.2196/58410

**Published:** 2025-03-27

**Authors:** Kaitlyn Hernandez-Spalding, Oluyemi Farinu, Lasha Clarke, Tamiah Lewis, Angie Suarez, Kimarie Bugg, Kieauna Strickland, Ashley Molleti, Sherry Maxy, Natalie Hernandez-Green

**Affiliations:** 1 Center for Maternal Health Equity Morehouse School of Medicine Atlanta, GA United States; 2 Reaching Our Sisters Everywhere Inc Lithonia, GA United States

**Keywords:** maternal health disparities, maternal near miss, minority health, mental health, narrative-based medicine, experiences, birthing experience, women, Black women, United States, maternal morbidity, patient-centered, racial, ethnic, disparities, socioeconomically, pregnancy, childbirth, postpartum, antenatal

## Abstract

**Background:**

In the United States, Black women are 3-4 times more likely to experience maternal near miss (MNM) or severe maternal morbidity (SMM) than non-Hispanic White women. However, there is a limited narrative-based investigation into Black and other marginalized women’s MNM experiences. Additionally, limited extant research on the impact of MNM and SMM on birthing women’s families or support persons and health care providers precludes the development of multilevel, patient-centered methods to eliminate these racial or ethnic disparities.

**Objective:**

This paper presents the protocol for a study that aims to draw insights from the experiences of racially and socioeconomically diverse mothers with MNM and SMM, their family or support persons (eg, partners), and health care providers to inform legislation, clinical practice, and infrastructure for optimal social support using PRISMA-P (Preferred Reporting Items for Systematic review and Meta-Analysis Protocols) guidelines. Using a storytelling approach to assess participants’ risk factors, document underlying causes, and research clinical causes of MNM, researchers hypothesize these data will inform policies to improve maternal conditions and provide safe and effective prevention and treatment options for birthing persons.

**Methods:**

Morehouse School of Medicine (MSM) will partner with health services and community-based organizations to promote inclusive participant recruitment for this multiphase study. In phase 1, qualitative interviews were conducted with birthing women (n≤87) who have experienced MNM and SMM. In phase 2, we will conduct qualitative interviews with the following groups: birthing women’s partners or support persons (n≤50), health care providers serving birthing women (n≤50), and adults who lost their mothers to pregnancy-related complications (n≤50). In each phase, the total number of participants interviewed will be based on theoretical saturation, that is, the point in iterative data collection and analysis when all important insights have been exhausted from the data already available.

**Results:**

Recruitment for phase 1 started in July 2021. As of March 2024, we have recruited 87 racially and socioeconomically diverse birthing women. Of those, 74% (64/87) self-identified as Black or African American, 20% (17/87) as Hispanic or Latina, and 9% (8/87) as Native American or Alaska Native. Severe preeclampsia accounted for 46% (40/87) of participants’ pregnancy-related adverse experiences. Qualitative interviews grounded in narrative-based medicine are ongoing. Recruitment for phase 2 will occur between July 2023 and December 2024. Study results will be published in peer-reviewed scientific journals.

**Conclusions:**

The findings from this research will deepen the understanding of how severe obstetric complications (1) are experienced by birthing women; (2) are perceived by their partners, support persons, and health providers; and (3) impact the lives of bereaved family and community members.

## Introduction

Maternal health encompasses the well-being of both mothers and their newborns during pregnancy, childbirth, and the postpartum period. Despite advancements in medical technology and health care systems, there remain persistent challenges with maternal mortality (MM) and morbidity. The Centers for Disease Control and Prevention reports that the MM rate for the United States in 2021 was 32.9 deaths per 100,000 live births, compared to a rate of 23.8 in 2020 and 20.1 in 2019—statistics that suggest MM is increasing [1]. Likewise, severe maternal morbidity (SMM) is also on the rise in the United States [2]. SMM involves unforeseen birth outcomes that cause short-term and long-term health effects for birthing persons, including hemorrhage, cardiac arrest, organ failure, major surgery, and other life-threatening complications that require interventions [3]. Sadly, disparities in adverse pregnancy-related outcomes are widening. Research shows women of color experience disproportionate rates of MM and SMM in comparison to their non-Hispanic White counterparts [4,5]. This issue is perpetuated by racism embedded within the maternal health care infrastructure to the extent of which social determinants, such as higher income and education, no longer serve as protective factors for health [5-9].

The World Health Organization (WHO) contends that maternal near miss (MNM), defined as “a woman who nearly died but survived a complication that occurred during pregnancy, childbirth or within 42 days of termination of pregnancy,” is a more useful indicator for studying the evaluation and improvement of obstetric health care than MM [10]. To date, little is understood about the contributors to MNM, especially for women and birthing people of color. Public health’s tendency to rely on medical records and statistics often renders those most affected by health disparities unseen, unheard, and unnoticed in the discourse [11]. As Silverio et al [12] wrote, “it is not uncommon for quantitative approaches to be unable to detect the nuances of the experiences we seek to understand.” Social context is needed to conceptualize the intricacies of health inequity as a means for developing effective and sustainable solutions [13]. Therefore, using a narrative-based medicine (NBM) model [14], this study centers on women of color’s lived experiences [15] with surviving life-threatening pregnancy complications. Additionally, we are collecting a multistakeholder perspective by interviewing health care providers and partners or support persons who have witnessed an MNM experience and gathering narratives from adults and caretakers of adults, who lost their birth mother due to maternal causes. Using this approach, we hope to obtain an understanding of these stakeholders’ perceptions and the impact of their experiences. Our goal is to uplift participants’ stories as data points for influencing maternal health legislation, clinical practice, and health care strategy.

## Methods

### Study Design and Conceptual Framework

This study explores the burden of MNM and SMM using a narrative or storytelling approach recognizing birthing persons’ experiences as legitimate sources of data. We used the Three Delays Model [16] to inform data collection and analysis. This model posits that MNM and SMM are largely the result of three critical delays, that are, first, delayed decision to seek care—barriers to making this critical decision include underestimation of the severity of the problem and its potential complications, poor understanding of danger signs and the potential scope of complications, cultural beliefs, customs, and attitudes (eg, distrust) regarding seeking care, and lack of social supports, among others. Second, delayed action or delay in reaching an appropriate site of care—getting to care, by definition, requires adequate transportation. A notorious problem in the developing world, transportation is also a challenge in many US states. Many states lack a sufficient number of perinatal providers; for example, half of Georgia’s 159 counties lack a maternity provider [17]. Barriers like health care insurance enrollment and coverage, provider network limitations, as well as financial constraints can also be difficult to navigate. Further, lack of social support and lack of personal agency may hinder a woman’s ability to act. Third, delayed diagnoses and appropriate treatment once a facility is reached—lack of facility resources (ie, equipment, blood, and drugs), a deficit of appropriately trained personnel, and systems that are poorly organized to manage obstetrical and medical emergencies are among the factors which can contribute to this delay.

### Eligibility and Recruitment

In phase 1, to be eligible for the study, participants had to meet the criteria of (1) self-identity as Black or African American, Indigenous, or Latinx; (2) older than 18 years of age; (3) meet WHO near miss criteria, that is, experience with severe postpartum hemorrhage, severe preeclampsia, eclampsia, sepsis or severe systemic infection, and ruptured uterus during pregnancy; and (4) can speak and read English. Additionally, the screener survey was modified to include specific questions about medical interventions participants may have experienced to differentiate between MNM and SMM experiences. Recruitment occurred from July 2021 through April 2022. States originally chosen for recruitment included Georgia, Louisiana, New Jersey, and the DMV area (Washington, DC; Maryland; and Virginia) due to their high rates of MM. In September 2021, New York, Connecticut, South Carolina, and Mississippi were added to increase recruitment, followed by the addition of Alabama, Texas, and Oklahoma in December 2021. After numerous inquiries from birthing persons outside the previously included states, the study was expanded to include the entire United States in March 2022.

In phase 2, health care providers must meet the criteria of (1) self-identify as a physician, nurse practitioner, physician assistant, midwife, pediatrician, psychologist, or doula; (2) over 50% of their patient population identifies as a racial or ethnic minority; and (3) had a patient that experienced an MNM or witnessed an MNM. Support persons or partners will be considered eligible if they meet the criteria of (1) self-identify as a support person for a birthing person who experienced an MNM; (2) self-identify as Black or African American, Indigenous, or Latinx; (3) older than 18 years of age; and (4) can read and speak English. For adults whose mothers died due to maternal causes and caretakers of adults whose mothers died due to maternal causes, participants are considered eligible if they meet the criteria of (1) the death of the mother must have occurred within 1 year of the adult’s birth; (2) self-identify as Black or African American, Indigenous, or Latinx; (3) older than 18 years of age; and (4) can read and speak English. An eligibility screener survey was created via REDCap (Vanderbilt University) for both phases of the study to identify participants.

### Data Collection

All internal and external team members were required to complete the basic Collaborative Institutional Training Initiative course. Team members were also trained in research interviewing techniques, including how to ask additional questions that may be relevant to each specific interview.

Before the interviews, each participant completed the screening survey to verify their eligibility. Participants also completed a voluntary survey collecting sociodemographic data in addition to data surrounding the structural determinants that may have contributed to their MNM experience. These factors include age, parity, marital status, place of residence, education level, income, neighborhood characteristics, food insecurity, and so forth. Scheduling for interviews took place over email or messages via the technology platforms provided by Optum. Interviews were conducted virtually over the Zoom platform (Zoom Video Communications), included both audio and video, and typically lasted between 1 and 2 hours.

Birthing persons were identified through contacts with Morehouse School of Medicine (MSM) partner organizations, including our national community partner Reaching Our Sisters Everywhere (ROSE). ROSE was founded to address breastfeeding disparities in Black communities and works to normalize breastfeeding by providing resources and networking opportunities for individuals and communities. As a national expert, and in partnership with communities, ROSE builds equity in maternal and child health and fatherhood initiatives through culturally appropriate training, education, advocacy, and support. This partnership served as an opportunity to combine our advocacy and support of the community. ROSE used its network to recruit participants and assisted in conducting interviews. MSM and ROSE team members closely supported one another in debriefing some of the challenging and emotionally charged conversations held with participants about their MNM experiences [12]. This study was supported by Optum, the health services business of United Health Group, through grant support and research participant recruitment. Screening criteria were the same across all tools, and participants were cross-referenced against past and scheduled participants to avoid duplication of data.

Consent for interview facilitation and recording and transcription of interviews in addition to the demographic information were all collected via REDCap. A detailed informed consent form was developed by the research team and approved by the MSM institutional review board (IRB). The consent form was completed during the Zoom session with a team member present to answer any questions prior to beginning the interview. After determining that mental health effects were a common theme during several interviews, the consent form was modified in the event that emergency professionals needed to be contacted. The adjusted language stated, “This certificate does not stop Morehouse School of Medicine from giving out information to prevent harm to you or others.” Any participants who mentioned suicidal ideation were also sent a Patient Safety Plan Template. This template, completed by individuals in their own time, collects warning signs, coping strategies, crisis contacts, and other material for participants to reference as needed [18]. Acknowledging that reliving traumatic experiences may have an effect, all participants received a detailed list of mental health resources located in their indicated state of residence.

Folders containing information about interviews and data analysis, including recruitment tracking, team interviewer availability, interview scheduling, and progress, were securely stored in an encrypted drive. Access was restricted to certified team members via password. Regular biweekly meetings were set up for the internal team to discuss updates and recruitment strategies. Additionally, separate biweekly working sessions were held with the funder.

As aforementioned, this study uses the power of storytelling, particularly NBM, which applies the narratives of patients or participants to medical practice [14]. We sought to understand participants’ interactions with health providers, perceptions of quality of care, the circumstances of their “near miss,” social support received, and their lived experiences prior to becoming pregnant. The interview guide was developed using the Three Delays Model and the International Consortium for Health Outcomes Measurement Set of Patient-Centered Outcome Measures for Pregnancy and Childbirth. These measures, including survival, morbidity, patient-reported health and well-being, and patient satisfaction with care were developed for providers to assess to improve patients’ health outcomes and well-being [19]. The interview guide was submitted to and approved by the MSM IRB. In total, the interview guide contained 12 main questions and 13 probing questions.

For example, 1 key question of the interview guide used the Three Delays Model:

How was the process when you arrived at the hospital and how was your complication resolved? Take me through this part. What was said to you? Did you know what was going on? What was communication like? How did you feel at that moment?

- Who/what were obstacles or facilitators to timely care?
- What was the wait time for care?
- Reasons for any delays
- Perceptions of quality of care

Furthermore, most of the interviews were spent answering the “near-miss” question:

Tell me about your birth experience. Tell me the story, all the way from beginning to end, describe the setting, who was involved, do you have any pictures you would like to share, please address important timelines...

After the completion of the interviews, each participant received an email with a US $100 gift card as compensation. Also included in the email was the list of mental health resources and a link to the screener if any participants wanted to share the study with others.

### Ethical Considerations

All Collaborative Institutional Training Initiative certificates were submitted and approved by the MSM IRB (ID number 1754465-15). Informed consent and the ability to opt out were provided to every participant. Participant data were deidentified. All participants who completed an interview were compensated with a US $100 gift card.

### Data Analysis

After interviews were transcribed using a transcription service, transcripts were uploaded into Dedoose, a web-based qualitative data analysis program developed by SocioCultural Research Consultants, LLC [20]. A qualitative analysis training session was conducted and recorded for all Optum and MSM team members involved in the process. Our team used an open coding approach in which the codes identified emerged from the data itself, also known as inductive coding [21,22]. Once codes were found, they were classified under larger themes to establish a codebook. Research team members met periodically to refine and collate codes. Each coded transcript was reviewed by another team member to ensure the consistency of the code application. If there were any disagreements regarding codes, team members were informed to bring it to the attention of the principal investigator, and a final code would be decided via a team discussion. Additionally, any suggestions for new codes were brought to the attention of the principal investigator. Data analysis began in May 2022 and was completed in March 2023. Coding included about 12-15 team members per round.

Our research question is qualitatively focused, though we will collect and analyze quantitative data in a few ways. We will use demographic and quantitative data first to comprehensively describe the study population. Second, we will use these data to explore whether qualitative themes vary across participant characteristics such as race, age, income, education, and the presence or absence of social support. As we iteratively review the interview data throughout the analysis process, we will also examine thematic differences across other relevant factors that may emerge. Finally, given there are adequate data to support these analyses, we will assess whether factors including demographic (eg, race, age, income, and education); psychosocial; and clinical (eg, receipt and timing of prenatal care) factors are associated type of pregnancy-related complications experienced. We will perform qualitative analyses with SAS (version 9.4; SAS Institute) and SPSS Statistics (version 29; IBM Corp) [23,24].

## Results

This study was funded in 2021 and recruitment for phase 1 started in July 2021. As of March 2024, we have recruited 87 racially and socioeconomically diverse birthing persons for phase 1. Of those, 74% (64/87) have self-identified as Black or African American, 20% (17/87) as Hispanic or Latina, and 9% (8/87) as Native American or Alaska Native (Table 1). Given the relatively low representation of some racial or ethnic groups, we will tailor ongoing recruitment efforts for phase 2 to improve inclusivity.

**Table 1:** Demographics of participants who completed an interview about the maternal near miss or severe maternal morbidity experience (N=87).

Variable			Values, n (%)
**Race and ethnicity**			
	Black or African American	64 (74)	
	Hispanic or Latino or Latina	17 (20)	
	Native American, Alaska Native	8 (9)	
	Asian	4 (5)	
	Middle Eastern	1 (1)	
	Indian	1 (1)	
	Hawaiian or Other Pacific Islander	0 (0)	
	White	0 (0)	
	Other	4 (5)	

Severe preeclampsia accounted for 46% (n=40) of participants’ pregnancy-related adverse experiences (Table 2). Qualitative interviews grounded in NBM are ongoing.

Recruitment for phase 2 is scheduled to occur between July 2023 and December 2024. Findings from each phase will be published in peer-reviewed scientific journals.

**Table 2:** Pregnancy-related complications that participants indicated experiencing (n=86).

Variable	Values, n (%)
Severe preeclampsia	40 (46)
Severe postpartum hemorrhage	22 (26)
Eclampsia	3 (4)
Ruptured uterus	2 (2)
Sepsis or severe systemic infection	1 (1)
Other	18 (21)

## Discussion

### Principal Findings

Collecting stories from our participants’ unique birthing experiences, as they relate to severe pregnancy-related complications, has allowed us to investigate the contributors to MNM and SMM and seek opportunities for improvement. Additionally, the majority of this study’s participants completed college or a graduate or professional degree and reported an annual household income of $50,000 or more. Therefore, gathering demographic data from participants provides insight into whether socioeconomic “protective” factors, including income and education, have a significant impact on a birthing person’s likelihood of experiencing MNM and SMM.

Sharing narratives from women of color who have experienced an MNM and SMM not only provides an opportunity to amplify the voices of those who have been historically silenced; but also, the evidence needed to advance maternal health justice. These perspectives are imperative in guiding the development of health priorities, policies, and strategies that drive optimal experiences for all birthing people. Some of our recommendations include equitable and respectful health care training, workforce diversification promotion, and health system disparity dashboards. Gathering stories from additional stakeholders will allow us to use their perspectives to refine our recommendations.

### Strengths and Limitations

Initially, the interviews conducted were determined to be experiences of SMM and MNM. Using the WHO near-miss approach, the screener survey was edited to include questions regarding critical interventions [10]. Participants were asked which critical interventions were performed to save their lives, including cesarean section, blood transfusion, and intensive care unit admission. After implementing these changes, the following interviews were determined to be the experiences of MNM. All SMM interviews were noted as such, and they were organized separately from the MNM interviews. Additionally, we noted many completed screener surveys were fraudulent. There was an influx of emails, in both the internal email account and our funder’s email account, that were spam and fraudulent. Screeners and emails were determined to be fraudulent if there were multiple screeners completed with different answers under the same email, the email addresses provided were invalid, and the open-ended answers or email communication did not grammatically make sense. To combat this issue, we incorporated a reCAPTCHA (Google) into our survey. Campbell et al [25] explain that humans and advanced bots can successfully avoid these mechanisms; this was consistent with our findings, given that reCAPTCHA did not seem to reduce the number of fraudulent screeners completed.

### Future Directions

At this time, the study has expanded to include partners and support persons of those who have experienced an MNM, health care providers who have witnessed an MNM, and adult children and their caretakers who lost their mothers due to maternal causes. Garnering a multistakeholder perspective about MNM, SMM, and maternal deaths will allow us to examine the impact that severe obstetric complications may have on family members, survivors, and health care providers.

## Abbreviations

IRB: institutional review board
MM: maternal mortality
MNM: maternal near miss
MSM: Morehouse School of Medicine
NBM: narrative-based medicine
PRISMA-P: Preferred Reporting Items for Systematic review and Meta-Analysis Protocols
ROSE: Reaching Our Sisters Everywhere
SMM: severe maternal morbidity
WHO: World Health Organization

## Data Availability

The datasets generated during and/or analyzed during this study are available from the corresponding author on reasonable request.
